# Impact of residential displacement on healthcare access and mental health among original residents of gentrifying neighborhoods in New York City

**DOI:** 10.1371/journal.pone.0190139

**Published:** 2017-12-22

**Authors:** Sungwoo Lim, Pui Ying Chan, Sarah Walters, Gretchen Culp, Mary Huynh, L. Hannah Gould

**Affiliations:** Division of Epidemiology, New York City Department of Health and Mental Hygiene, Queens, New York, United States of America; University of Miami, UNITED STATES

## Abstract

**Objectives:**

As gentrification continues in New York City as well as other urban areas, residents of lower socioeconomic status maybe at higher risk for residential displacement. Yet, there have been few quantitative assessments of the health impacts of displacement. The objective of this paper is to assess the association between displacement and healthcare access and mental health among the original residents of gentrifying neighborhoods in New York City.

**Methods:**

We used 2 data sources: 1) 2005–2014 American Community Surveys to identify gentrifying neighborhoods in New York City, and 2) 2006–2014 Statewide Planning and Research Cooperative System. Our cohort included 12,882 residents of gentrifying neighborhoods in 2006 who had records of emergency department visits or hospitalization at least once every 2 years in 2006–2014. Rates of emergency department visits and hospitalizations post-baseline were compared between residents who were displaced and those who remained.

**Results:**

During 2006–2014, 23% were displaced. Compared with those who remained, displaced residents were more likely to make emergency department visits and experience hospitalizations, mainly due to mental health (Rate Ratio = 1.8, 95% confidence interval = 1.5, 2.2), after controlling for baseline demographics, health status, healthcare utilization, residential movement, and the neighborhood of residence in 2006.

**Conclusions:**

These findings suggest negative impacts of displacement on healthcare access and mental health, particularly among adults living in urban areas and with a history of frequent emergency department visits or hospitalizations.

## Introduction

Residential displacement is a likely consequence of severe housing cost burden, which is defined by the United States Department of Housing and Urban Development as spending 50% or more of one’s income on housing costs [1]. Previous studies have shown that residential displacement may disrupt existing social ties, increase stress, and reduce social and economic resource availability [2, 3]. These changes have been associated with adverse health outcomes. For example, a study in Chicago showed that displaced residents from public housing had higher levels of stress and depression [3]. A recent cohort study in Dallas, Texas also found that moving to more deprived neighborhoods was associated with weight gain [4].

One of the possible external drivers of displacement among urban residents is gentrification. Gentrification is a process through which deprived neighborhoods are revitalized by economic development, typically resulting in an influx of new residents of higher socioeconomic status [5]. While gentrification has positive socioeconomic impacts, such as increased access to supermarkets and business opportunities [5], it may also lead to increased housing and rent burden, which can in turn result in displacement of residents who originally resided in gentrifying neighborhoods [2, 6, 7]. A study in Philadelphia compared persons who moved from gentrifying neighborhoods with those who moved from non-gentrifying neighborhoods; the former were more likely to end up in more disadvantaged neighborhoods [8].

In New York City (NYC), a quarter of neighborhoods underwent gentrification between 1990 and 2014 [6]. As gentrification continues in NYC, residents of lower socioeconomic status may be at high risk of displacement and suffer from increased physical and mental health burden and disrupted healthcare access, exacerbating health inequities. Yet, there have been few systematic, quantitative assessments of whether displacement from gentrifying neighborhoods is associated with healthcare utilization and health outcomes. Large, longitudinal datasets are needed to describe movements of original residents over the course of gentrification and to investigate the association of displacement with health outcomes.

In this study, we used large administrative datasets of emergency department (ED) visits and hospitalization events in NYC hospitals, and followed the residents who resided in gentrifying neighborhoods in 2006. Of these, we defined residents who moved from gentrifying to non-gentrifying, poor neighborhoods as displaced. We then assessed the association between displacement and ED visits and hospitalizations during 2006–2014. Because displacement might lead to disrupted access to primary care services, we tested the hypothesis that displacement is associated with increased ED visits and hospitalizations. Given a potential pathway between displacement and increased stress, we also tested a hypothesis that displacement is associated with increased mental health-related ED visits and hospitalizations.

## Materials and methods

We used 2 data sources: 1) 2005–2014 American Community Survey (ACS) data to identify gentrifying and non-gentrifying, poor neighborhoods in NYC, and 2) 2006–2014 Statewide Planning and Research Cooperative System (SPARCS) to obtain records of ED visits and hospitalizations and assess the association between displacement and ED visits and hospitalizations among residents of gentrifying neighborhoods.

### Definition of gentrifying and non-gentrifying neighborhoods

We defined gentrifying and non-gentrifying neighborhoods in NYC, based in part on variables used in other studies [9, 10]. We obtained single-year estimates of median household income, median rental price, and proportion of adults aged ≥25 years with a college degree for each NYC neighborhood from 2005–2014 ACS data. Neighborhoods were defined using Public Use Microdata Area (PUMA) boundaries (n = 55, median population in each PUMA = 149,447 according to 2014 ACS). We ranked neighborhoods (range: 1–55) by each of the 3 characteristics in 2005 and by the degree of linear growth on each characteristic during 2005–2014. Using these 6 rankings, we defined gentrifying neighborhoods as neighborhoods with low initial rankings (i.e., low median household income, median rental price, and proportion of college graduates in 2005) and high rankings of growth (i.e., rapid increase in median household income, median rental price, and proportion of college graduates). Non-gentrifying, poor neighborhoods were defined as neighborhoods with low initial and growth rankings. We used principal component analysis (PCA) (described later) to identify neighborhoods meeting these definitions.

### Study population

SPARCS is a data reporting system that collects discharge information, such as patients’ demographics and clinical data, from all hospitals in New York State. We geocoded patients’ residential addresses with an algorithm that used NYC Geosupport Desktop Edition and USPS verification. This systematic approach allowed for efficiently geocoding a large number of addresses and identifying those who lived in gentrifying NYC neighborhoods in 2006 and had at least 1 geocodable NYC address every 2 years (i.e., they were hospitalized or visited an ED in NYC at least once every 2 years) during 2006–2014 (N = 18,472). Of this cohort, we considered those who had ever moved to a non-gentrifying, poor neighborhood after 2006 as displaced residents (N = 3,376). We compared these individuals (displaced group) with those who remained in gentrifying neighborhoods (comparison group 1) (N = 11,731). In some instances a comparison person moved; however, if the person remained in the same type of neighborhood for the entire study period, he/she was included in the analysis. We excluded individuals under 18 years of age at baseline (defined later). We also excluded individuals who had ≥3 different addresses in any one of the years before baseline to remove persons who might have been homeless. The final sample consisted of 2,937 displaced residents and 9,945 individuals in comparison group 1. As a sensitivity analysis, we also compared displaced persons with those who lived in non-gentrifying, poor neighborhoods (comparison group 2) during 2006–2014 (N = 9,227).

For the displaced group, we defined baseline (i.e., time point of displacement) as the midpoint between the date of the 1^st^ ED visit or hospitalization with a non-gentrifying neighborhood address and the date of the previous visit. Because displacement did not occur in the comparison groups, we defined their baseline as the average baseline of the displaced group, which was January 4, 2010.

### Measures

The study outcomes included post-baseline (from baseline through 2014) counts of ED visits, hospitalizations, and mental health-related visits (ED visits and hospitalizations combined). We identified mental health-related visits using the Clinical Classification Software category of the primary diagnosis. Covariates included baseline age (18–24, 25–44, 45–64, 65+), sex, pre-baseline (from 2006 to baseline) counts of ED visits per year, pre-baseline counts of hospitalizations per year, and number of residential movements during the year before baseline (0, 1, >1). Additionally, because individuals’ health conditions were a strong predictor of ED visits and hospitalizations and could introduce confounding, we included as covariates whether a person had ever had a pre-baseline primary diagnosis of any of the 15 Clinical Classification Software categories (S1 Table).

The NYC Department of Health and Mental Hygiene Institutional Review Board determined that the current study was an exempt activity.

### Statistical analysis

#### Principal component analysis

We conducted PCA to capture variance in the neighborhood-level characteristics (i.e., initial rankings and growth rankings in median household income, median rent, and proportion of college graduates) across NYC neighborhoods. This dimension reduction technique allowed us to explain total variances by linear combinations of these 6 neighborhood rankings (i.e., principal components) and graphically describe neighborhoods that shared similar characteristics. Principal components were ordered by the amount of explained variance (i.e., a linear combination that explained the largest variance is labeled as the first principal component). The first 2 principal components explained 78% of the total variance, which sufficiently summarized the data. The first principal component represented the 3 initial rankings, and the second principal component captured the 3 rankings in growth. Using each principal component as an axis, we plotted NYC neighborhoods in a biplot and identified groups of NYC neighborhoods that met the definitions of gentrifying and non-gentrifying neighborhoods (S1 Fig). Specifically, we considered a neighborhood gentrifying if the first PCA loading score was less than the bottom 80^th^ percentile and the second PCA loading score was greater than the top 25^th^ percentile. To identify non-gentrifying, poor neighborhoods, we selected neighborhoods in the bottom 25^th^ percentile of the first PCA and the bottom 80^th^ percentiles of the second PCA loading scores. We finalized neighborhood selections by examining the actual rankings of the baseline characteristics and their change during the study period.

#### Inverse probability of treatment weight

To address possible differences in the underlying demographic characteristics, health status, and healthcare utilization between displaced and comparison groups, we estimated the probability of displacement using logistic regression with displacement as an outcome and baseline characteristics (described in Measure) as covariates. We then constructed inverse probability of treatment weights (IPTW) by inverting propensity scores and stabilized them by replacing the numerator with a marginal distribution of displacement (i.e., distribution of displacement in the overall population) to reduce influences from large weights [11]. After IPTW were incorporated, data met the causal inference assumptions for exchangeability (balanced baseline characteristics between displaced and comparison groups), positivity (tightly distributed IPTW with 1 as a mean value), and stable unit treatment value (after adjustment for the original residential neighborhood to address potential data dependency among those who lived in the same neighborhood) [12]. Because there were two separate comparison groups, we created 2 sets of IPTW.

#### Regression analysis

We created 6 separate negative binomial regression models to compare the post-baseline rate of ED visits, hospitalizations, and mental health-related visits, between displaced persons and each of the 2 comparison groups. To address residual confounding that might not be addressed by IPTW (i.e., remaining imbalance after IPTW), we included the following covariates in the regression models: age group, sex, counts of visits (restricted to the same type of visits as the model outcome) during 1 year before baseline, and number of residential movements during 1 year before baseline. This produced doubly robust estimates that performed better than other propensity score methods in terms of reducing bias due to confounding and variance estimation [13]. To address potential clustering of persons who lived in the same neighborhood, we included the neighborhood of residence in 2006 as an additional covariate [14]. Lastly, to address potential model misspecification, we estimated variance using a Generalized Estimation Equation.

To understand whether the post-baseline hospital visits were different in nature between displaced residents and residents who stayed in gentrifying neighborhoods, we conducted a post-hoc analysis to describe the primary diagnoses of all post-baseline visits. We examined diagnoses related to ambulatory care sensitive (ACS) conditions (e.g., asthma, congestive heart failure, diabetes, hypertension) because ACS condition-related visits are preventable admissions and may indicate insufficient primary care, which was one of the hypotheses tested in this study [15]. We also examined specific diagnoses related to mental illness. Lastly, we quantified bias due to unobserved confounding such as race/ethnicity and socioeconomic factors using the bias equation of Vanderweele and Arah and assessed its impact on the rate ratios of visits by displacement status [16].

In order to understand whether gentrification per se was associated with health outcomes, we performed an additional sensitivity analysis where we assessed differences in baseline characteristics between the 2 comparison groups and repeated the regression analysis. We hypothesized that residents living in the gentrifying neighborhoods were more likely to visit ED and/or get hospitalized than residents living in non-gentrifying, poor neighborhoods if gentrification itself had a negative impact on health.

All analyses were performed using SAS 9.4 (SAS Institute, NC) except for PCA, which was performed using R 3.2.0 (R Foundation for Statistical Computing, Vienna, Austria). A 2-sided p-value < 0.05 was considered statistically significant.

## Results

**Fig 1:**
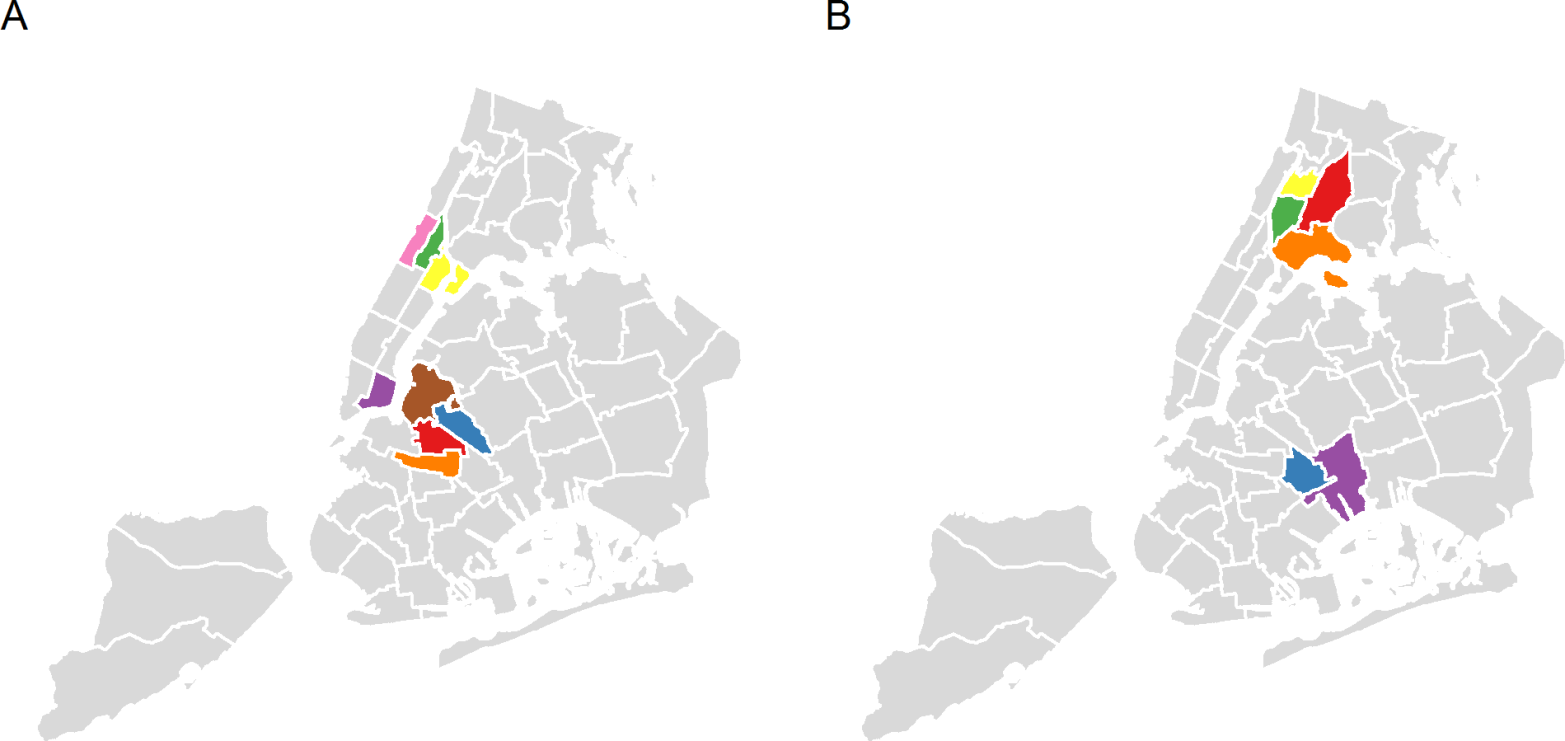
Gentrifying and non-gentrifying, poor neighborhoods, New York City, 2005–2014. Legend: (A) Gentrifying neighborhoods included Crown Heights North and Prospect Heights (orange color); Bedford-Stuyvesant (red color); Chinatown and Lower East Side (purple color); Bushwick; Greenpoint and Williamsburg (blue color); Hamilton Heights and West Harlem (pink color); Central Harlem (green color); and East Harlem (yellow color). (B) Non-gentrifying, poor neighborhoods included East New York and Starrett City (purple color); Morris Heights and Fordham South (yellow color); Brownsville and Ocean Hill (blue color); Hunts Point, Longwood and Melrose (orange color); Belmont, Crotona Park East, and East Tremont (red color); and Concourse, High bridge and Mount Eden (green color). Notes: Gentrifying neighborhoods were defined as having a low ranking in 2005 in median household income, median gross rental price, and proportion of adults aged 25 or older with a college degree, and a high ranking in the growth of these variables during 2005–2014; non-gentrifying, poor neighborhoods were defined as having a low ranking in the same set of variables in 2005 and a low ranking in the growth.

During 2005–2014, 47 of the 55 neighborhoods in NYC experienced growth in all 3 variables we analyzed (i.e., median household income, median rental price, and proportion of college graduates). Based on PCA and our examination of the actual rankings, 8 neighborhoods were identified as gentrifying, including 4 Manhattan (Chinatown & Lower East Side, Hamilton Heights & West Harlem, Central Harlem, East Harlem) and 4 Brooklyn neighborhoods (Crown Heights North & Prospect Heights, Bedford-Stuyvesant, Bushwick, Greenpoint & Williamsburg) (Fig 1). Six neighborhoods were classified as non-gentrifying, poor neighborhoods, including 2 Brooklyn (East New York & Starrett City, Brownsville & Ocean Hill) and 4 Bronx neighborhoods (Morris Heights & Fordham South, Hunts Point, Longwood & Melrose, Belmont, Crotona Park East & East Tremont, Concourse, Highbridge & Mount Eden) (Fig 1).

Compared with the general NYC adult population in 2010 [17], the original residents of the gentrifying neighborhoods in this study consisted of a higher proportion of women (72% vs. 54%) and persons aged 45–64 years (37% vs. 28%). The proportions of the original residents with mental illness (22% vs. 21%) and diabetes (9% vs. 9%), based on primary diagnosis, were similar to those among the NYC population, while the proportion of those with health insurance was higher (93% vs. 83%) [17, 18]. Most baseline characteristics were significantly different between displaced and comparison groups before IPTW (Table 1). Compared with residents who remained in gentrifying neighborhoods during 2006–2014 (n = 9,945), displaced residents (n = 2,937) were more likely to be men (35% vs. 26%), young adults (18–44 years at baseline) (66% vs. 42%), and move at least once in the year before baseline (20% vs. 3%) (Table 1). Displaced residents also had more ED visits (yearly average: 2.6 vs. 2.1), hospitalizations (yearly average: 0.9 vs. 0.7), and were more likely to be diagnosed with mental health related conditions (37% vs. 18%) before baseline. Similar differences were observed when comparing displaced residents with residents who stayed in non-gentrifying, poor neighborhoods (data not showed). After IPTW, the distribution of age, number of hospital visits, number of residential movements, and clinical characteristics became similar between displaced and comparison groups; the sex distribution remained different.

**Table 1:** Baseline characteristics of displaced residents of gentrifying neighborhoods and residents who remained in the neighborhoods, New York City, 2006–2014.

Characteristics	Before IPTW		After IPTW	
	Displaced residents	Residents who remained	Displaced residents	Residents who remained
	N = 2,937	N = 9,945	N = 2,937	N = 9,945
	% or mean (SD)			
Age group				
18–24	**18.2**	**9.7**	12.0	11.9
25–44	**47.6**	**32.7**	37.0	36.1
45–64	**30.2**	**38.4**	35.4	36.3
65+	**4.0**	**19.2**	15.7	15.7
Sex				
Female	**64.9**	**74.4**	**70.2**	**72.2**
Male	**35.1**	**25.6**	**29.8**	**27.8**
Mean number of pre-baseline ED visits per year ^a^	**2.6 (2.5)**	**2.1 (2.4)**	2.3 (2.0)	2.3 (3.5)
Mean number of pre-baseline hospitalizations per year ^a^	**0.9 (1.4)**	**0.7 (1.1)**	0.8 (0.8)	0.8 (0.8)
Number of residential movements in the year before baseline ^b^				
0	**79.6**	**96.7**	92.7	92.8
1	**11.5**	**2.1**	4.3	4.2
>1	**8.9**	**1.2**	3.0	3.0
Pre-baseline diagnoses (ever) ^a^^,^^c^				
Infectious and parasitic diseases	**25.2**	**22.2**	**25.5**	**23.2**
Neoplasms	**3.2**	**5.2**	5.3	4.8
Endocrine, nutritional, metabolic, and immunity disorders	**12.0**	**15.5**	16.1	14.6
Blood disorders	**2.5**	**3.3**	3.6	3.2
Nervous system diseases	**37.2**	**42.6**	**44.5**	**41.7**
Circulatory system diseases	**30.7**	**42.1**	40.6	39.6
Respiratory diseases	**53.9**	**59.8**	**61.7**	**58.7**
Digestive diseases	**38.6**	**42.0**	**44.7**	**41.6**
Genitourinary diseases	37.2	37.1	39.4	37.4
Complications of pregnancy, childbirth, and puerperium	**24.8**	**14.4**	17.8	16.9
Musculoskeletal and skin diseases	**52.6**	**60.8**	60.3	59.1
Congenital and perinatal diseases	0.2	0.4	0.3	0.3
Injury and poisoning	52.3	52.3	**54.8**	**52.4**
Mental illness	**36.6**	**18.2**	24.0	22.4
Other	52.0	51.5	**54.6**	**51.9**

Note: Boldface indicates statistical significance (p<0.05); IPTW, Inverse probability of treatment weight; SD, Standard Deviation.^a^From 2006 to baseline (the time point of displacement)^b^Based on the number of unique addresses recorded^c^Proportion of persons with a primary diagnosis of the corresponding category.

On average, the cohort was followed up for 5 years post-baseline. Compared with residents who remained in the gentrifying neighborhoods, displaced residents had significantly higher rates of ED visits (rate ratio [RR] = 1.1, 95% confidence interval [CI] = 1.0–1.2, p = 0.005), hospitalizations (RR = 1.3, 95% CI = 1.2–1.4, p<0.001), and mental health-related visits (RR = 1.8, 95% CI = 1.5–2.2, p<0.001), controlling for baseline demographics, health status, healthcare utilization, residential movement, and the neighborhood of residence in 2006 (Table 2). In a sensitivity analysis comparing displaced residents with residents of non-gentrifying, poor neighborhoods (Table 2), similar results were observed. Displacement was significantly associated with increased ED visits (RR = 1.2, 95% CI = 1.1–1.2, p<0.001), hospitalizations (RR = 1.2, 95% CI = 1.1–1.3, p<0.001), and mental health-related visits (RR = 1.7, 95% CI = 1.4–2.0, p<0.001).

**Table 2:** Rate ratio of emergency department visits, hospitalizations, and mental health-related visits among displaced residents of gentrifying neighborhoods versus comparison groups, New York City, 2006–2014.

	Emergency department visits		Hospitalizations		Mental health-related visits	
	Rate ratio^a^ (95% confidence interval)	p-value	Rate ratio^a^ (95% confidence interval)	p-value	Rate ratio^a^ (95% confidence interval)	p-value
Displaced residents vs. residents who remained in gentrifying neighborhoods	**1.1 (1.0, 1.2)**	0.005	**1.3 (1.2, 1.4)**	<0.001	**1.8 (1.5, 2.2)**	<0.001
Displaced residents vs. residents who continuously lived in non-gentrifying neighborhoods	**1.2 (1.1, 1.2)**	<0.001	**1.2 (1.1, 1.3)**	<0.001	**1.7 (1.4, 2.0)**	<0.001

Note: Boldface indicates statistical significance (p<0.05).

^a^Negative binomial model with inverse probability of treatment weight was used. Results for displaced residents vs. residents who remained in gentrifying neighborhoods were controlled for age, sex, history of health care utilization and residential movements, and neighborhood of residence in 2006. The same covariates were included the model for displaced residents vs. residents who continuously lived in non-gentrifying neighborhoods except for neighborhood of residence in 2006, which caused a complete separation problem.

Displaced residents did not have a higher proportion of post-baseline visits related to ACS conditions compared with residents who stayed in gentrifying neighborhoods (20% vs. 25%). However, the proportion of alcohol-related admissions among displaced residents was about 6 times that of the comparison group (10% vs. 2%). In addition, the proportion of drug-related admissions among displaced residents was about 7 times that of the comparison group (4% vs. 0.6%). Given a substantially higher proportion of visits related to alcohol and drug issues among displaced residents, we excluded alcohol- and drug-related visits from the outcomes and re-ran the regression analysis. Despite a reduced magnitude of the association (ED visits: RR = 1.1, 95% CI = 1.0–1.1; hospitalizations: RR = 1.2, 95% CI = 1.1–1.3; mental health visits: RR = 1.4, 95% CI = 1.1–1.6), the results remained statistically significant. Lastly, we tested the extent to which the RR of mental health visits was biased due to unobserved confounding. The RR of mental health visits remained statistically significant even after accounting for unobserved confounding (S2 Fig).

According to the additional sensitivity analysis, we found generally similar baseline characteristics between residents who remained in gentrifying neighborhoods and those who remained in non-gentrifying, poor neighborhoods. Residents of gentrifying neighborhoods, as opposed to those of non-gentrifying, poor neighborhoods, had significantly higher rates of ED visits (RR = 1.1, 95% CI = 1.0–1.1), but lower rates of hospitalizations (RR = 0.95; 95% CI = 0.91–0.98). The rates of mental health-related visits were not significantly different between these two groups. (RR = 1.0; 95% CI = 0.9–1.1).

## Discussion

In this study, we identified adults who lived in gentrifying NYC neighborhoods in 2006 and found that those who moved to non-gentrifying, poor neighborhoods had a greater number of ED visits, hospitalizations, and mental health-related visits for about 5 years after displacement. These findings suggest negative impacts of displacement on healthcare access and mental health, particularly among adults living in densely populated urban areas and with a history of frequent ED visits or hospitalizations.

The significant impact of displacement on mental health observed in this study is consistent with previous studies where displaced individuals were assessed for mental health conditions after demolition of public housing units [3] or natural disasters and conflict [19, 20]. Although the causes of displacement may be vastly different, it may be “root shock” that links displacement with mental health [21, 22]. Displaced persons no longer have access to the same social networks, may lose community ties, and suffer disruptions in regular routines, which increase stress and psychological distress [19, 21, 23]. The impact of displacement on mental health may differ based on the housing outcome. In a study examining the experience of residents of New Orleans following Hurricane Katrina, residents who had to relocate following Hurricane Katrina and could not return to their original community experienced higher general psychological distress and perceived stress as compared with residents who were able to return [19]. Residents who were unstably housed and experienced multiple moves had the highest levels of perceived stress [19]. Similarly, a study in Baltimore found that moving more than twice during a six month period was associated with depressive symptoms [24]. Although our study assessed displacement from gentrifying neighborhoods, it is likely that the inability to return to one’s community and the loss of social networks may have a similar impact on mental health.

We also found that displaced residents were more likely to make ED visits or be hospitalized for alcohol- and drug-related issues. One possible explanation is that increased stress from displacement might lead to increased alcohol or drug intake [25]. Another possible explanation is that individuals with preexisting alcohol or substance use problems might be more susceptible to displacement. However, we believe that the latter explanation is less probable because we controlled for history of mental health diagnoses and frequency of moving before displacement, and the association between displacement and mental health remained even after excluding alcohol- and drug-related visits.

Increased ED use and hospitalizations among displaced residents of gentrifying neighborhoods might reflect disruptions in access to primary healthcare. During the period of dislocation and re-settlement, displaced persons may use ED as a primary healthcare resource [26, 27]. In a national survey, living in areas with high residential instability was associated with poorer access to care (i.e., no usual source of care, ED as a usual source of care, or unmet medical needs) [26]. In another study of NYC mothers receiving public assistance, a recent history of moving was associated with reporting the ED as a usual source of care [27]. Alternatively, this association might be explained by greater exposure to environmental hazards such as poor housing stock and hazardous indoor pollutants, which may be more prevalent in non-gentrifying, poor neighborhoods than gentrifying neighborhoods and increase susceptibility to stress [28, 29].

Our sensitivity analysis showed that rates of mental health-related visits were not different between residents who remained in gentrifying neighborhoods and those who remained in non-gentrifying, poor neighborhoods. This suggests that gentrification per se might not be associated with health outcomes. However, we believe that the pathway of gentrification leading to displacement, which in turn impacts healthcare utilization, is possible. In a post-hoc analysis, we found that a higher proportion of residents from gentrifying neighborhoods in 2006 moved to non-gentrifying, poor neighborhoods during 2006–14, compared with those from other neighborhoods (i.e., other than non-gentrifying, poor neighborhoods) (17.7% vs 13.2%). Further studies are needed to validate this potential pathway and examine the reasons for displacement.

The main strength of this study was the innovative use of administrative data to create a cohort of individuals, assess residential movements, and characterize neighborhood-level changes. Another strength was that our quantitative approach to identify gentrifying neighborhoods aligned with qualitative and anecdotal information about gentrification in NYC. The gentrifying neighborhoods we identified were a subset of those defined by the New York University Furman Center in an analysis of gentrification in NYC across 25 years [6].

Our analysis had several limitations. First, we do not know the reasons for moving from gentrifying to non-gentrifying neighborhoods; it is possible these movements were due to factors unrelated to gentrification. Second, there are limited identifiers in SPARCS and it is possible that we matched some persons incorrectly. Third, while we excluded persons who were likely homeless based on ≥3 different addresses in a 1-year period, some homeless persons might still remain in the analysis. Fourth, SPARCS contains billing data and very few sociodemographic variables. Despite a good balance in baseline demographic and clinical characteristics between the displaced and comparison groups, it is possible that some residual confounding due to unknown factors remained. In particular, given the small magnitude of the rate ratios, the observed impact of displacement on ED visits or hospitalizations might disappear if additional confounders are controlled for. However, as seen in the sensitivity analysis, the rate ratio of mental health visits remained statistically significant regardless of various values of unobserved confounding. Fifth, these findings might have limited external validity because the study cohort consisted of adults living in NYC who frequently visited EDs or experienced hospitalizations. Despite limited generalizability, the cohort members were unlikely to represent those in extremely poor health conditions given that their prevalence of mental illness and diabetes at baseline was similar to that of the general NYC population. Sixth, because single-year ACS data do not produce reliable estimates at geographies smaller than PUMA, we classified neighborhoods at the PUMA level, and it is possible that smaller geographies within a PUMA were misclassified as gentrifying or non-gentrifying neighborhoods. Lastly, we did not differentiate neighborhoods based on the rate or timing of gentrification. It is possible that displaced residents from neighborhoods of earlier versus later stage of gentrification might be different from the others. Future studies are warranted to examine how the association between displacement and health outcomes may differ across neighborhoods with different rates or timing of gentrification.

In conclusion, this study is one of the first to directly quantify the relationship of residential displacement on health. We identified a large cohort of persons who visited an ED or were hospitalized frequently and were displaced, and found that moving was associated with increased ED visits and hospitalizations, particularly for mental health-related conditions. This analysis helps to identify groups of people who might be more susceptible to displacement from gentrifying neighborhoods. Strengthening systems for mental health support and services might help to prevent ED visits and hospitalizations among displaced persons. In NYC, programs like Thrive NYC (thrivenyc.cityofnewyork.us) are strengthening mental health services for all New Yorkers at need. In addition, active engagement and collaboration between local governments and community-based organizations will help raise awareness of negative impacts of displacement and implement programs to reduce displacement (e.g., rent freeze program, affordable units). These types of programs are essential in light of the rapid changes underway in many NYC neighborhoods, and will help protect health of residents who are vulnerable to displacement.

As gentrification has reshaped physical, social, and economic characteristics of neighborhoods, it is critical for public health practitioners to assess how this neighborhood-level change impacts the health of original residents, especially those who are vulnerable to displacement. Future studies are needed to further assess the dynamic relationship between neighborhood and health and to strengthen public health systems to monitor and assess the impact of gentrification on health.

## Supporting information

S1 Fig Biplot from principal component analysis using American Community Survey data, New York City, 2005–2014. This figure illustrates where each New York City neighborhood is located in terms of initial rankings and growth rankings in median household income, median rent, and proportion of college graduates.
(DOCX)

S2 Fig Estimated lower bound of 95% confidence intervals of rate ratio of mental health visits by displacement adjusted for an unobserved confounder (U). This figure illustrates how lower bound of 95% confidential intervals of rate ratio of mental health visits by displacement is affected by various scenarios of unobserved confounding.
(DOCX)

S1 Table A list of 15 Clinical Classification Software categories. This table lists descriptions of 15 Clinical Classification Software categories that we included in the propensity score models.
(DOCX)

## References

[1] U.S. Department of Housing and Urban Development. Rental burdens: rethinking affordability measures. 2014. https://www.huduser.gov/portal/pdredge/pdr_edge_featd_article_092214.html. Accessed November 14, 2017.

[2] Freeman L, Branconi F. Gentrification and displacement: New York City in the 1990s. J Am Plann Assoc 2004;70(1):39–52.

[3] Keene DE, Geronimus AT. “Weathering” Hope IV: the importance of evaluating the population health impact of public housing demolition and displacement. J Urban Health 2011;88(3):417–435. doi: 10.1007/s11524-011-9582-5 21607787 10.1007/s11524-011-9582-5 PMC3126923

[4] Powell-Wiley TM, Cooper-McCann R, Ayers C, Berrigan D, Lian M, McClurkin M, et al Change in neighborhood socioeconomic status and weight gain: Dallas heart study. Am J Prev Med 2015;49(1):72–79. doi: 10.1016/j.amepre.2015.01.013 25960394 10.1016/j.amepre.2015.01.013 PMC4476924

[5] Kennedy M, Leonard P. Dealing with Neighborhood Change: A Primer on Gentrification and Policy Choices. The Brookings Institution Center on Urban and Metropolitan Policy and PolicyLink. 2001 http://www.policylink.org/sites/default/files/DealingWithGentrification_final.pdf. Accessed April 5, 2017.

[6] NYU Furman Center. State of New York City Housing and Neighborhoods in 2015. May 2015. http://furmancenter.org/files/sotc/NYUFurmanCenter_SOCin2015_9JUNE2016.pdf. Accessed May 26, 2017.

[7] Marcuse P. Gentrification, abandonment, and displacement: connections, causes, and policy responses in New York City. J Urban Contemp Law 1985;28:195–240.

[8] Ding L, Hwang J, Divringi E. Gentrification and residential mobility in Philadelphia. Reg Sci Urban Econ 2006;61:38–51. 10.1016/j.regsciurbeco.2016.09.004 PMC5450830 28579662

[9] Steinmetz-Wood M, Wasfi R, Parker G, Bornstein L, Caron J, Kestens Y. Is gentrification all bad? Positive association between gentrification and individual’s perceived neighborhood collective efficacy in Montreal, Canada. Int J Health Geogr 2017; 16(1):24 doi: 10.1186/s12942-017-0096-6 28709431 10.1186/s12942-017-0096-6 PMC5513321

[10] Gibbons J, Barton MS. The Association of Minority Self-Rated Health with Black versus White Gentrification. J Urban Health 2016; 93(6):909–22. doi: 10.1007/s11524-016-0087-0 27761683 10.1007/s11524-016-0087-0 PMC5126023

[11] Robins JM, Hernán MA, Brumback B. Marginal structural models and causal inference in epidemiology. Epidemiology 2000;11(5):550–560. 10955408 10.1097/00001648-200009000-00011

[12] Cole SR, Hernán MA. Constructing inverse probability weights for marginal structural models. Am J Epidemiol 2008;168(6):656–664. doi: 10.1093/aje/kwn164 18682488 10.1093/aje/kwn164 PMC2732954

[13] Austin PC. The performance of different propensity-score methods for estimating differences in proportions (risk differences or absolute risk reductions) in observational studies. Stat Med 2010;29(20):2137–48. doi: 10.1002/sim.3854 20108233 10.1002/sim.3854 PMC3068290

[14] Kasza J, Polkinghorne KR, Marshall MR, McDonald SP, Wolfe R. Clustering and residual confounding in the application of marginal structural models: dialysis modality, vascular access, and mortality. Am J Epidemiol 2015;182(6):535–43. doi: 10.1093/aje/kwv090 26316597 10.1093/aje/kwv090

[15] Billings J, Zietel L, Lukomnik J, Carey TS, Blank AE, Newman L. Impact of socioeconomic status on hospital use in New York City. Health Aff (Millwood) 1993;12:162–173. 10.1377/hlthaff.12.1.162 8509018

[16] Vanderweele TJ, Arah OA. Bias formulas for sensitivity analysis of unmeasured confounding for general outcomes, treatments, and confounders. Epidemiology 2011;22(1): 42–52. doi: 10.1097/EDE.0b013e3181f74493 21052008 10.1097/EDE.0b013e3181f74493 PMC3073860

[17] New York City Department of Health and Mental Hygiene. Epiquery: NYC Interactive Health Data System—Community Health Survey 2009. Available at: http://nyc.gov/health/epiquery. Accessed 5/9/2017.

[18] New York City Department of Health and Mental Hygiene. Epiquery: NYC Interactive Health Data System–New York City Health and Examination Survey 2013–14. Available at: http://nyc.gov/health/epiquery. Accessed 5/24/2017.

[19] Fussell E, Lowe SR. The impact of housing displacement on the mental health of low-income parents after Hurricane Katrina. Soc Sci Med 2014;113:137–144. doi: 10.1016/j.socscimed.2014.05.025 24866205 10.1016/j.socscimed.2014.05.025 PMC4096953

[20] Fazel M, Reed RV, Panter-Brick C, Stein A. Mental health of displaced and refugee children resettled in high-income countries: risk and protective factors. Lancet 2012;379(9812):266–282. doi: 10.1016/S0140-6736(11)60051-2 21835459 10.1016/S0140-6736(11)60051-2

[21] Fullilove MT. *Root shock*: *How tearing up city neighborhoods hurts America*, *and what we can do about it*: One World/Ballantine; 2009.

[22] Fullilove MT. Root shock: the consequences of African American dispossession. J Urban Health 2001;78(1):72–80. doi: 10.1093/jurban/78.1.72 11368205 10.1093/jurban/78.1.72 PMC3456198

[23] Cetner for Disease Controla and Prevention. Health Effects of Gentrification. Available at: https://www.cdc.gov/healthyplaces/healthtopics/gentrification.htm. Accessed 5/4/17, 2017.

[24] Davey-Rothwell M, German D, Latkin CA. Residential transience and depression: does the relationship exist for men and women? J Urban Health 2008;85(5):707–716. doi: 10.1007/s11524-008-9294-7 18581237 10.1007/s11524-008-9294-7 PMC2527435

[25] Keyes KM, Hatzenbuehler ML, Hasin DS. Stressful life experiences, alcohol consumption, and alcohol use disorders: the epidemiologic evidence for four main types of stressors. Psychopharmacology (Berl) 2011;218(1):1–17. 21373787 10.1007/s00213-011-2236-1 PMC3755727

[26] Kirby JB, Kaneda T. Access to health care: does neighborhood residential instability matter? J Health Soc Behav 2006;47(6):142–155. doi: 10.1177/002214650604700204 16821508 10.1177/002214650604700204

[27] Dunchon LM, Weitzman BC, Shinn M. The relationship of residential instability to medical care utilization among poor mothers in New York City. Med Care 1999;37(12);1282–1293. 10599609 10.1097/00005650-199912000-00011

[28] Krieger J, Higgins DL. Housing and health: time again for public health action. Am J Public Health 2002;92(5):758–768. 11988443 10.2105/ajph.92.5.758 PMC1447157

[29] Clougherty JE, Kubzansky LD. A framework for examining social stress and susceptibility to air pollution in respiratory health. Environ Health Perspect 2009;117(9):1351–1358. doi: 10.1289/ehp.0900612 19750097 10.1289/ehp.0900612 PMC2737009

