# Supplementary material for: Impact of residential displacement on healthcare access and mental health among original residents of gentrifying neighborhoods in New York City
Source: PLoS One. 2017 Dec 22;12(12):e0190139. doi: 10.1371/journal.pone.0190139 (PMC5741227; doi:10.1371/journal.pone.0190139)
Supplement: S1 Fig — This figure illustrates where each New York City neighborhood is located in terms of initial rankings and growth rankings in median household income, median rent, and proportion of college graduates. (DOCX) [file pone.0190139.s001.docx]

**S1 Fig. Biplot from Principal Component Analysis Using American Community Survey Data, New York City, 2005-2014**


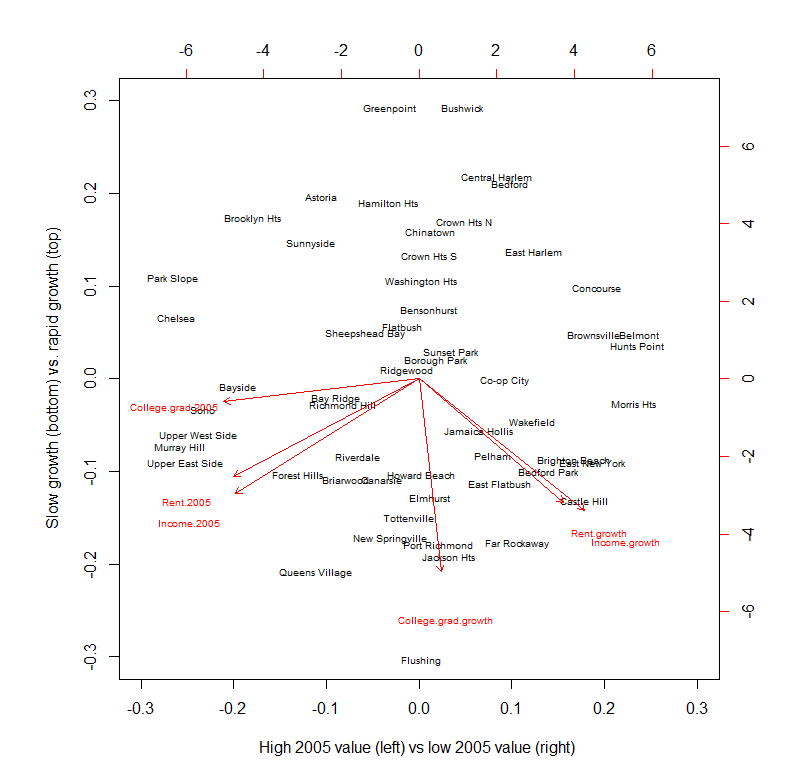


Notes: The neighborhoods circled in blue color were defined as gentrifying neighborhoods because they had a low ranking in 2005 in median household income, median gross rental price, and proportion of adults with a college degree, and a high ranking in the growth of these variables during 2005-2014. The neighborhoods circled in green color were defined as non-gentrifying, poor neighborhoods due to their low rankings of both initial characteristics and growth.
