# Supplementary material for: Impact of residential displacement on healthcare access and mental health among original residents of gentrifying neighborhoods in New York City
Source: PLoS One. 2017 Dec 22;12(12):e0190139. doi: 10.1371/journal.pone.0190139 (PMC5741227; doi:10.1371/journal.pone.0190139)
Supplement: S2 Fig — This figure illustrates how lower bound of 95% confidential intervals of rate ratio of mental health visits by displacement is affected by various scenarios of unobserved confounding. (DOCX) [file pone.0190139.s002.docx]

**S2 Fig. Estimated Lower Bound of 95% Confidence Intervals of Rate Ratio of Mental Health visits by Displacement Adjusted for an Unobserved Confounder (U)**

Notes: The lower bound of 95% CI of rate ratio of mental health visit by displacement was 1.4 from Table 2. *P(U=1|a=1,x)* was arbitrarily set as 0.5.

*U* = unmeasured confounder; *δ = P(U=1|a=1, x) / P(U=1|a=0,x)*; *γ* = *RR* of mental health visits by *U*.
