# Supplementary material for: Impact of residential displacement on healthcare access and mental health among original residents of gentrifying neighborhoods in New York City
Source: PLoS One. 2017 Dec 22;12(12):e0190139. doi: 10.1371/journal.pone.0190139 (PMC5741227; doi:10.1371/journal.pone.0190139)
Supplement: S1 Table — This table lists descriptions of 15 Clinical Classification Software categories that we included in the propensity score models. (DOCX) [file pone.0190139.s003.docx]

**S1 Table. A list of 15 Clinical Classification Software categories**

| CCS diagnosis number | Description |
| --- | --- |
| 1-10 | Infectious and parasitic diseases |
| 11-47 | Neoplasms |
| 48-58 | Endocrine, nutritional, metabolic, and immunity disorders |
| 59-64 | Blood disorders |
| 76-95 | Nervous system diseases |
| 96-121 | Circulatory system diseases |
| 122-134 | Respiratory diseases |
| 135-155 | Digestive diseases |
| 156-175 | Genitourinary diseases |
| 176-196 | Complications of pregnancy, childbirth, and puerperium |
| 197-212 | Musculoskeletal and skin diseases |
| 213-224 | Congenital and perinatal diseases |
| 225-244 | Injury and poisoning |
| 650-663, 670 | Mental illness |
| 245-260 | Other |
